# Huge Overlap of Individual TCR Beta Repertoires

**DOI:** 10.3389/fimmu.2013.00466

**Published:** 2013-12-25

**Authors:** Mikhail Shugay, Dmitriy A. Bolotin, Ekaterina V. Putintseva, Mikhail V. Pogorelyy, Ilgar Z. Mamedov, Dmitriy M. Chudakov

**Affiliations:** ^1^Shemyakin-Ovchinnikov Institute of Bioorganic Chemistry, Russian Academy of Science, Moscow, Russia; ^2^Central European Institute of Technology (CEITEC), Masaryk University, Brno, Czech Republic

**Keywords:** adaptive immunity, TCR repertoire, TCR beta, NGS data analysis, overlap

It has been reported that human TCR repertoires commonly carry so-called public clonotypes – CDR3 variants that are often shared between individuals. Cross-comparison of individual immune repertoires has previously revealed the existence of a population of TCR beta CDR3 variants that are identical at the amino acid level for any two donors (1–3). The lower bound for the total overlap between any two given donors’ TCR beta repertoires within their CD8+ naïve T cell subset has been estimated as ~14,000 identical amino acid CDR3 variants based on comparison of 200,000–600,000 individual TCR beta clonotypes (1). Here, we have used deep profiling data consisting of 1–2 × 10^6^ individual TCR beta clonotypes that we obtained from healthy donors (4) to better estimate the total overlap between TCR beta repertoires for any two individuals.

**Figure 1:**
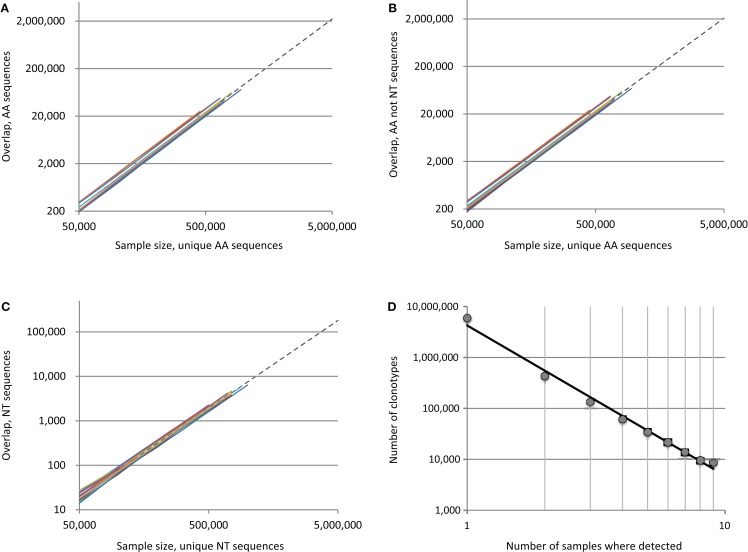
**Overlap of individual TCR beta CDR3 repertoires grows geometrically with the number of sequence pairs sampled**. Plots indicate the number of shared sequences for 12 unrelated donor pairs in relation to sample size at the level of **(A)** all amino acid sequences, **(B)** amino acid sequence only, excluding matches with identical nucleotide sequences, and **(C)** nucleotide sequences. Each of the 12 colored lines represents the observed overlap between randomly drawn samples of unique CDR3 variants for a different pair of unrelated donors. To extrapolate the predicted level of overlap if the full individual TCR beta repertoires were to be sampled, we plotted fittings of averaged data with a power law (*Y* = *aX^b^*) as dashed lines. **(D)** We plotted the degree to which unique clonotypes were shared among our nine donors, and found that the frequency with which TCR beta clonotypes occur in human repertoires is distributed according to a power law.

The apparent paradox is, that the deeper we sequence, the larger is the percentage of observed overlapping clonotypes between the two repertoires, since the number of possible element pairs between the two sets grows geometrically. To demonstrate this, we analyzed TCR beta repertoires for 12 unrelated pairs assembled from a total of nine human donors [adults and children, see Ref. (4) for details]. We plotted the number of identical variants found in samples of increasing size, with up to 10^6^ unique CDR3 sequences randomly drawn from the repertoires of each individual in a given pair (Figure 1). For every pair, the number of shared clonotypes grew geometrically with the arithmetic growth of the sample size (Figures 1A–C, colored lines); at maximum sequencing depth (~1 × 10^6^ unique sequences/donor), we observed an average of ~72,000, 68,000, and 6,000 CDR3 variants that were respectively identical at the amino acid, amino acid only/non-nucleotide and nucleotide level. This exceeds previous estimates (1) by several-fold. The greatest overlap was between two donors from whom we obtained ~1 × 10^6^ and 1.7 × 10^6^ CDR3 variants, where we observed 113,000, 108,000, and 11,000 identical clonotypes at the amino acid, amino acid only/non-nucleotide and nucleotide level, respectively.

The lower bound on total individual TCR beta repertoire diversity has previously been estimated to be 5 × 10^6^ unique clonotypes [Ref. (5) and our unpublished data]. With that in mind, we extrapolated our intersection curves by fitting them to a power law model [*Y* = *aX^b^*, as in Ref. (1)], which yielded coefficient “*b*” close to 2.0 and *R*^2^ > 0.999 for all cases (Figures 1A–C, dashed lines). We estimated that the total overlap of the TCR beta CDR3 repertoires for two individuals constitutes ~2,200,000, 2,060,000, and 180,000 variants, i.e. 44.1, 41.3, and 3.6% of a given individual’s sequence diversity at the amino acid, amino acid only/non-nucleotide, and nucleotide level, respectively.

Thus, the real paradox is that nearly half of the TCR beta CDR3 repertoire is functionally identical between any two individuals, in spite of the fact that the theoretical diversity that can be achieved by TCR beta variants has been estimated to be ~5 × 10^11^ sequences (1, 6). The results from our extrapolation are direct and evident. We took numerous precautions to exclude contamination in our work, including sequencing of pair-analyzed donor repertoires in separate Illumina lanes (4). Even if contaminations were present, these would not affect overlap at the amino acid only/non-nucleotide level (Figure 1B). Furthermore, we performed CDR3 extraction and error correction with MiTCR (http://mitcr.milaboratory.com/) using the stringent ETE algorithm, which eliminates 98% of PCR and sequencing errors with minimal loss of natural TCR beta diversity (7).

Such large overlap between individuals suggests the existence of a rather limited pool of frequently used functional CDR3 sequences. To further investigate this, we calculated the lower and upper bounds of the Chao richness estimate as described in Ref. (8) based on the numbers of singletons and doubletons (sequences observed in one and two individuals, respectively) in 12 paired donors’ samples. From this model, we obtained a confidence interval of 1.2 × 10^7^ to 5.4 × 10^7^ unique amino acid CDR3 sequences, at a significance level of α = 0.001.

These findings represent a shift in our understanding of human adaptive immunity. It now appears likely that recombinatorial biases (3, 9) and thymic selection (4, 10, 11) shape our repertoires so tightly that the majority of TCR beta CDR3 variants expressed by naïve T cells leaving the thymus are chosen from a “short-list” of just under 10^8^ amino acid variants – even shorter than the 2 × 10^9^ “effective sequence space” estimated by Robins and colleagues (1).

Nevertheless, the repertoire has a complex structure and those clonotypes that are characterized as low-complexity [see figure 7 in Ref. (4)] predominantly form the backbone of the shared clonotype pool. Interestingly, when we examined the intersection of all nine donor samples, we found that the number of donors in which a given clonotype can be detected is distributed according to a power law, with a degree of −2.95 and *R*^2^ = 0.99 (Figure 1D). These findings confirm the fractal structure of the human TCR beta repertoire that determines the landscape of shared clonotypes (1–3, 12), and may reveal a more complex picture with the deeper profiling experiments.
